# Insight into the molecular mechanism behind PEG-mediated stabilization of biofluid lipases

**DOI:** 10.1038/s41598-018-29871-z

**Published:** 2018-08-16

**Authors:** Bianca Pérez, Andrea Coletta, Jannik N. Pedersen, Steen V. Petersen, Xavier Periole, Jan Skov Pedersen, Richard B. Sessions, Zheng Guo, Adam Perriman, Birgit Schiøtt

**Affiliations:** 1 Department of Engineering, Aarhus University, Aarhus, 8000 Denmark; 2 Department of Chemistry, Aarhus University, Aarhus, 8000 Denmark; 3 Interdisciplinary Nanoscience Center, Aarhus University, Aarhus, 8000 Denmark; 4 Department of Biomedicine, Aarhus University, Aarhus, 8000 Denmark; 5 School of Biochemistry, University of Bristol, Bristol, BS8 1TD UK; 6 School of Cellular and Molecular Medicine, University of Bristol, Bristol, BS8 1TD UK

## Abstract

Bioconjugates established between anionic polyethylene glycol (PEG) based polymers and cationic proteins have proven to be a promising strategy to engineer thermostable biocatalysts. However, the enzyme activity of these bioconjugates is very low and the mechanism of non-covalent PEG-stabilization is yet to be understood. This work presents experimental and molecular dynamics simulation studies, using lipase-polymer surfactant nanoconjugates from mesophile *Rhizomucor miehei* (RML), performed to evaluate the effect of PEG on enzyme stability and activity. Results demonstrated that the number of hydrogen bonds between the cationized RML and PEG chain correlates with enzyme thermostability. In addition, an increase of both the number of PEG-polymers units and cationization degree of the enzyme leads to a decrease of enzyme activity. Modelling with SAXS data of aqueous solutions of the biofluid lipases agrees with previous hypothesis that these enzymes contain a core constituted of folded protein confined by a shell of surfactants. Together results provide valuable insight into the mechanism of non-covalent PEG mediated protein stabilization relevant for engineering active and thermostable biofluids. Furthermore, the first biofluids RML with activity comparable to their cationized counterpart are presented.

## Introduction

Enzymatic catalysis has made major contributions to clean manufacturing and green processes. However, the low stability of enzymes restricts their extensive application^1^. Site-directed mutagenesis is one technique used to promote enzymes resistance to environmental stress^2^. However, generic engineering of stable enzymes is highly time consuming and expensive. Alternatively, straightforward chemical modification can be performed to reactive groups present in a protein to yield a more stable form of the enzyme^3,4^. In fact, recent reports have demonstrated that the modification of aspartic and glutamic amino acid residues of enzymes using carbodiimide crosslinker chemistry and *N,N*′-dimethyl-1,3-propanediamine (DMPA), supports the formation of cationic proteins that have the capacity to form non-covalent interactions with anionic polymer surfactants, producing thermostable non-covalent enzyme complexes^5–7^ (here referred to as biofluid enzymes). Although biofluid enzymes reported up-to-date present poor catalytic activity, these conjugates represent new doors for the development of novel enzyme technology as they challenged the view that a hydration shell is required for an enzyme to be stable^7–9^. Thus, it becomes of great interest for the scientific and industrial community to understand the enzyme-polymer surfactant interactions to enhance enzyme thermostability without hampering enzyme activity.

Different classes of proteins have been used to form protein-polymer surfactant nanoconjugates^6,7,10^, including lipases. Lipases are among the most investigated biocatalysts used in a wide range of application from organic synthesis to industrial production of functional lipids (e.g., Lipozyme TL IM catalyzed production of margarine). The structure of lipases generally has a polypeptide chain lid that inhibits access to the catalytic triad. However, in the presence of a hydrophobic substance, it is displaced, and hence allows substrate access to the catalytic site^11^. Depending on the reaction conditions, lipases may both catalyze the hydrolysis of triglycerides and drive esterification. One lipase that presents high activity under diverse conditions (supercritical fluids and organic solvents, etc.), but which is limited by its poor thermostability, is the lipase from the mesophile *Rhizomucor miehei* (RML)^12^. Although synthesis of biofluid RML using a cationized enzyme with 60% efficiency and an oxidized version of BrijL23 (Synonym: Brij 35, C12E23, Polyoxyethylene (23) lauryl ether) generated a more thermostable enzyme than its unmodified counterpart, the enzyme activity towards *p-*nitrophenyl butyrate was nearly lost^7^.

Though several studies have been carried out to investigate the physicochemical properties of biofluid enzymes and demonstrate the scientific significance of this emerging technology, few reports describe the non-covalent interaction of these anionic polymer surfactants with the cationized enzyme and accessibility of the substrate to the enzyme^13,14^. In the present work, we address both issues by combining experimental results with molecular dynamics (MD) simulations. The molecular dynamics simulation were carried out based on previous reports were 60% of cationization efficiency was achieved for RML^7^. Thus, the 3D structures used for MD simulations were designed by modifying 60% of ASP and GLU of RML using the DMPA structure. In the experimental work, preparation of cationized RML was performed at low temperature to avoid thermal stress, prevent modification of the catalytic triad, and potentially lead to a more active biofluid form of the enzyme. DMPA was covalently coupled to 45% of the side chain of the ASP and GLU present in the enzyme using EDC (1-ethyl-3-(3-dimethylaminopropyl)carbodiimide) and NHS (N-hydroxysuccinimide) as coupling reagents. The cationized enzyme was then made interact through non-covalent bonds with the different anionic surfactants (S2 = glycolic acid ethoxylate lauryl ether, or S7 = oxidized Brij L23, containing 11–13 or 23 monomer units of ethylene glycol, correspondingly) to yield the corresponding biofluids. The resulting biofluid enzymes were characterized in the pure form (without solvent) by Fourier transform infrared spectroscopy (FTIR), and in aqueous solution by circular dichroism (CD) in the Far-UV. Furthermore, small-angle X-ray scattering (SAXS) measurements were carried out to obtain information regarding the shape, conformation, and assembly state of the different macromolecules studied. In addition, the enzymatic activity of these enzymes was evaluated by measuring the hydrolysis of *p*-nitrophenyl palmitate. The results combined provide unique information to (i) describe the interaction of the cationized lipase with different anionic polymer surfactants; (ii) identify structural features relevant to confer thermostability to the enzyme, and (iii) evaluate the substrate accessibility to the catalytic triad. Furthermore, as far as we know, this work presents the first MD simulations describing the non-covalent interaction of cationized lipases with polymer surfactant and the biofluid RML’s structure resulted from modelling with SAXS data. In addition, as predicted the low temperature of the cationization reaction led to a lower cationization degree (approximately 45% of the ASP and GLU of the enzymes were modified compared to previous reports that showed that 60% of the ASP and GLU of the enzymes were modified^7^) and consequently, generated more active biofluids.

## Results and Discussion

### Synthesis of novel biofluid lipases

**Figure 1:**
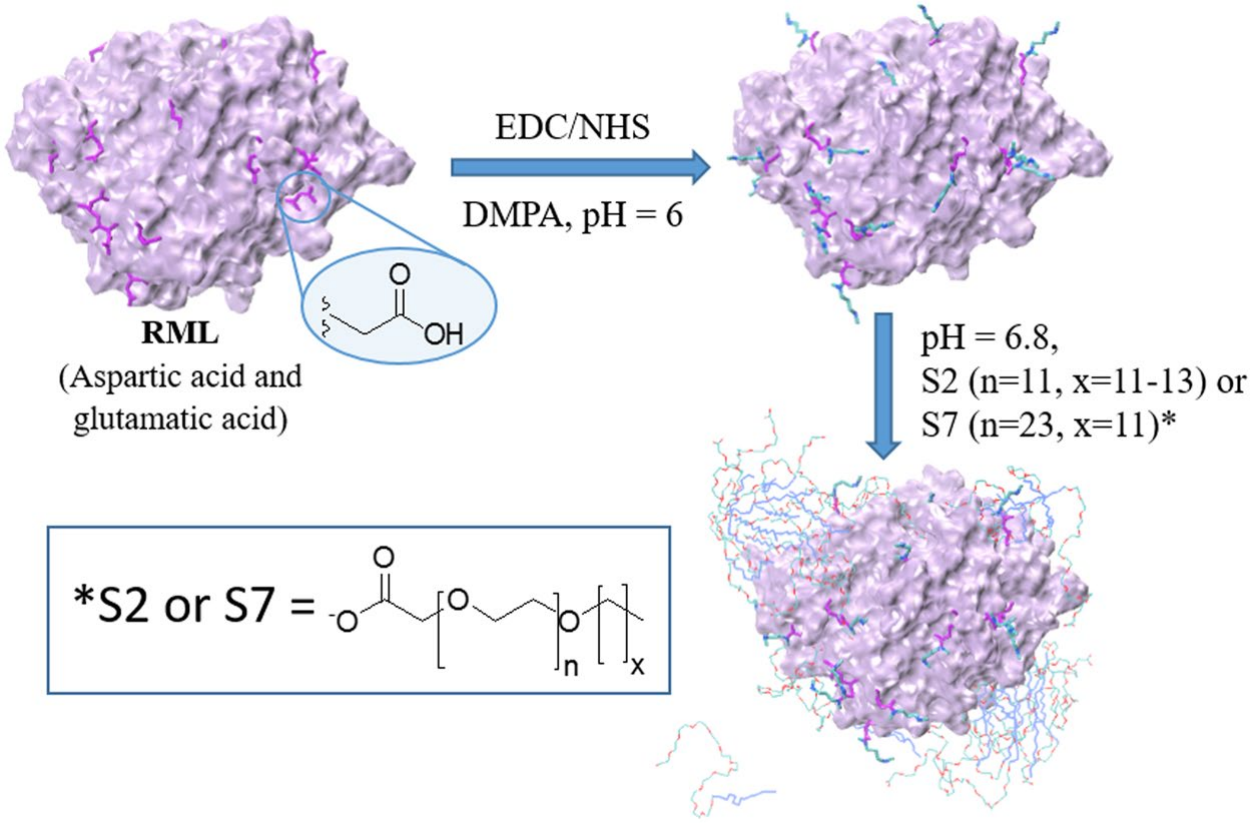
Protocol followed to prepare biofluids RML using EDC (1-ethyl-3-(3-dimethylaminopropyl)carbodiimide), NHS (N-hydroxysuccinimide), and *N,N*′-dimethyl-1,3-propanediamine (DMPA), and polymer surfactants (S2 and S7). Protein, amino acid side chain, modified side chain and polymer are represented in MSMS, purple Licorice, Licorice colored by atom, and Lines, respectively.

Asp and Glu residues of RML (solid form) were cationized using DMPA and carbodiimide crosslinker chemistry (Fig. 1) and S2 and S7 were respectively non-covalently associated to the resulting enzyme following a procedure similar to one previously described^7^. However, the cationization reaction was performed <4 °C instead to avoid thermal stress on the lipase, potentially prevent modification of the active site and yield a more active form of the biofluid enzyme. Thus, lower cationization efficiency was achieved (45% of the enzyme was cationized; cRML45) compared to previous studies were enzyme cationization was performed at room temperature (60% of the enzyme was cationized; cRML60)^7^. The cationization efficiency was calculated based on MALDI-TOF MS (Fig. S1). Subsequently, cRML45 was modified by the addition of anionic surfactants (S2 and S7, respectively) and the sample containing the conjugates were dialyzed for 24 hours against water with the aim of removing the excess of surfactants. To confirm the presence of the surfactant in the dried powders obtained after dialysis and freeze-dried, FTIR was used to characterize RML, cRML45, and cRML45-S2, and cRML45-S7, S2, and S7 in their pure form (without solvent; Fig. S2). FTIR spectra showed that all lipase variants displayed the characteristic peaks of a protein including the signal of the N-H stretching around 1620 cm^−1^ and only cRML45-S2 and cRML45-S7 among the lipases tested showed the C-H stretching between 2850–2900 cm^−1^ and the C-O stretching around 1110 cm^−1^ characteristic of the S2 and S7 polymer surfactants. The latter demonstrated that the desired complexes were formed. Thus, the molar ratio of polymer to cationized enzyme was calculated using Bicinchoninic acid (BCA) protein assay. Based on results cRML45:S2 and cRML45:S7 present a molar ratio of 1:205 and 1:146, respectively. Since approximately 12 ASP and GLU in the enzyme were functionalized and the enzyme contains 7 LYS and 10 ARG (i.e. Total number of binding sites for cRML45 equals 29), an excess of polymer in relationship to binding site remains in the samples after dialysis. Further characterization of the conjugates in aqueous solution is presented below.

### Circular dichroism in the far-UV of the new biofluid lipases

**Figure 2:**
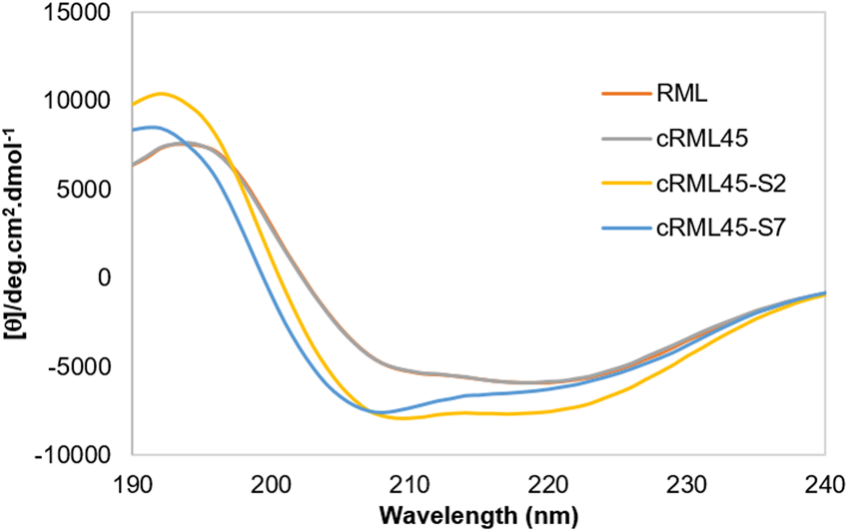
CD spectra from 190 nm to 240 nm of the lipase variants in aqueous solution and at room temperature. Measurements were done in the absence of buffer. RML analyzed was obtained after dialysis and freeze-drying.

CD spectroscopy can provide information regarding the secondary structure content and the thermostability of an enzyme. Hence, all the lipases were characterized by CD spectroscopy. Figure 2 shows the CD spectra from 190 to 240 nm of each of the lipase variants, and the secondary structure content was calculated using Dichroweb web server (Table 1)^15,16^. This analysis suggests that all enzymes displayed a high content of β-sheet structure (>24%) and that the cationization of RML does not significantly influence its secondary structure. For instance, both RML and cRML45 presented similar α-helix (16%) and turns content (19%) and only a small difference in the β-sheet (28% and 29%, correspondingly) and random coil content (35% and 36%, respectively). However, the interaction of the anionic polymer surfactant with the cationized enzymes causes a slight drop in the β-sheet content in the case of cRML45-S2, which correlates with an increase in the α-helix content. However, no significant differences were observed between cRML45-S7 and the unmodified enzyme as cRML45-S7 displayed only a small increase of α-helix content. The difference observed between cRML45-S2 and cRML45-S7 could be attributed to the length of the PEG chain as the longer PEG chain of S7 could potentially confer more steric stabilization than S2. Furthermore, cRML45-S7’s results slightly differ from values previously reported for cRML60-S7 where an increase of the β-sheet content and an decrease of random coil was observed instead^7^. This discrepancy could be attributed to the different cationization degree between the cationized enzyme previously reported (~60%,) and the cationized RML here presented (~45%).

**Table 1:** Secondary structure content of RML, cRML45, and biofluid Lipases calculated from CD spectroscopy measurements from 240 nm to 190 nm in aqueous conditions using Dichroweb web server.

Enzyme	α-helix	β-sheet	Turns	Random coil	NRMSD
RML*	16	28	19	36	0.026
cRML45	16	29	19	35	0.021
cRML45-S2	21	24	18	35	0.016
cRML45-S7	18	28	19	35	0.020

*RML analyzed was obtained after dialysis and freeze-drying.

**Figure 3:**
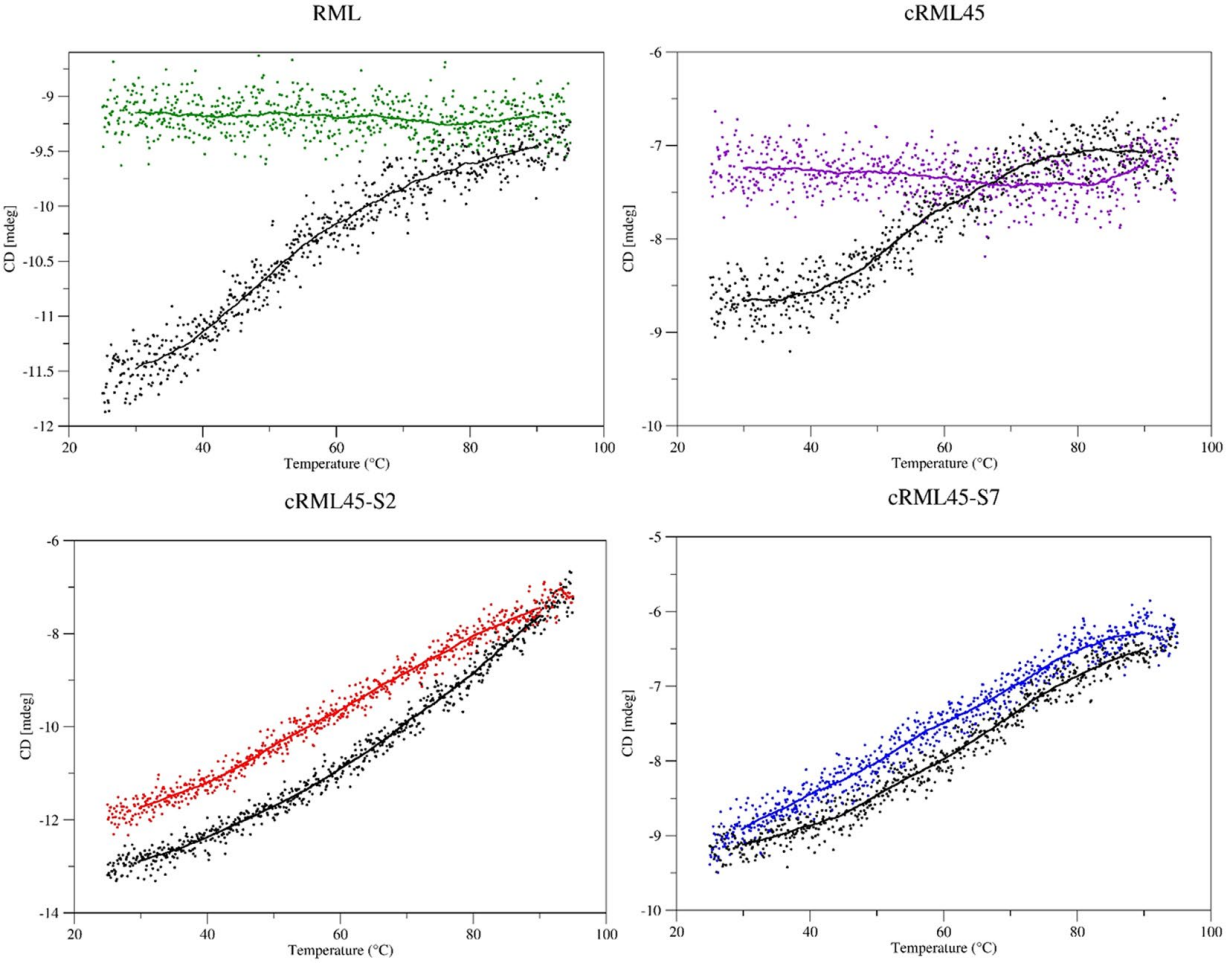
Plot of the CD signal at 222 nm from 25 °C to 95 °C (Black) and from 95 °C to 25 °C (Colored) of the lipase variants in aqueous conditions in the absence of buffer. Heating rate used was 5 °C per min. RML analyzed was obtained after dialysis and freeze-drying.

To evaluate the thermostability of the enzymes, the CD spectroscopy signal at 222 nm was monitored over a thermal cycle between 25 °C and 95 °C (Fig. 3). The results showed that both RML and cRML45 were unable to recover their secondary structure when the system was heated to 95 °C and cooled down to 25 °C. On the contrary, cRML45-S2 and cRML45-S7 were capable of recovering their secondary structure. However, a slight difference was observed between the cRML45-S2 and cRML45-S7, as the former did not display identical starting and end points, contrary to cRML45-S7. This suggests slightly higher thermostability in the case of cRML45-S7; possibly, due to the longer PEG chain present in S7. The higher thermostability of cRML45-S7 is also reflected when measuring the CD spectra from 190 to 250 nm before and after temperature scan, and 95 °C (Fig. S3) as cRML45-S7 did not completely loss the secondary structure at 95 °C, contrary to results found for cRML45-S2. Similar conclusions were drawn for PEGylated proteins synthesized using different activated monomethoxypolyethylene glycol (mPEG)s (Mw 1100, 2000 and 5000 g/mol); they observed that when the molecular weight of the mPEG increased, the covalent conjugates became more stable^17,18^. Thus, the size of PEG is a key factor determining the thermostability of both covalent and non-covalent conjugates. The ability of the PEG chain to form hydrogen bond with the surface of the protein and displace water molecules could prevent enzyme aggregation and explain the higher thermostability observed for the biofluids when comparing to the unmodified enzyme.

### Small-angle X-ray scattering of the new biofluid lipases

**Figure 4:**
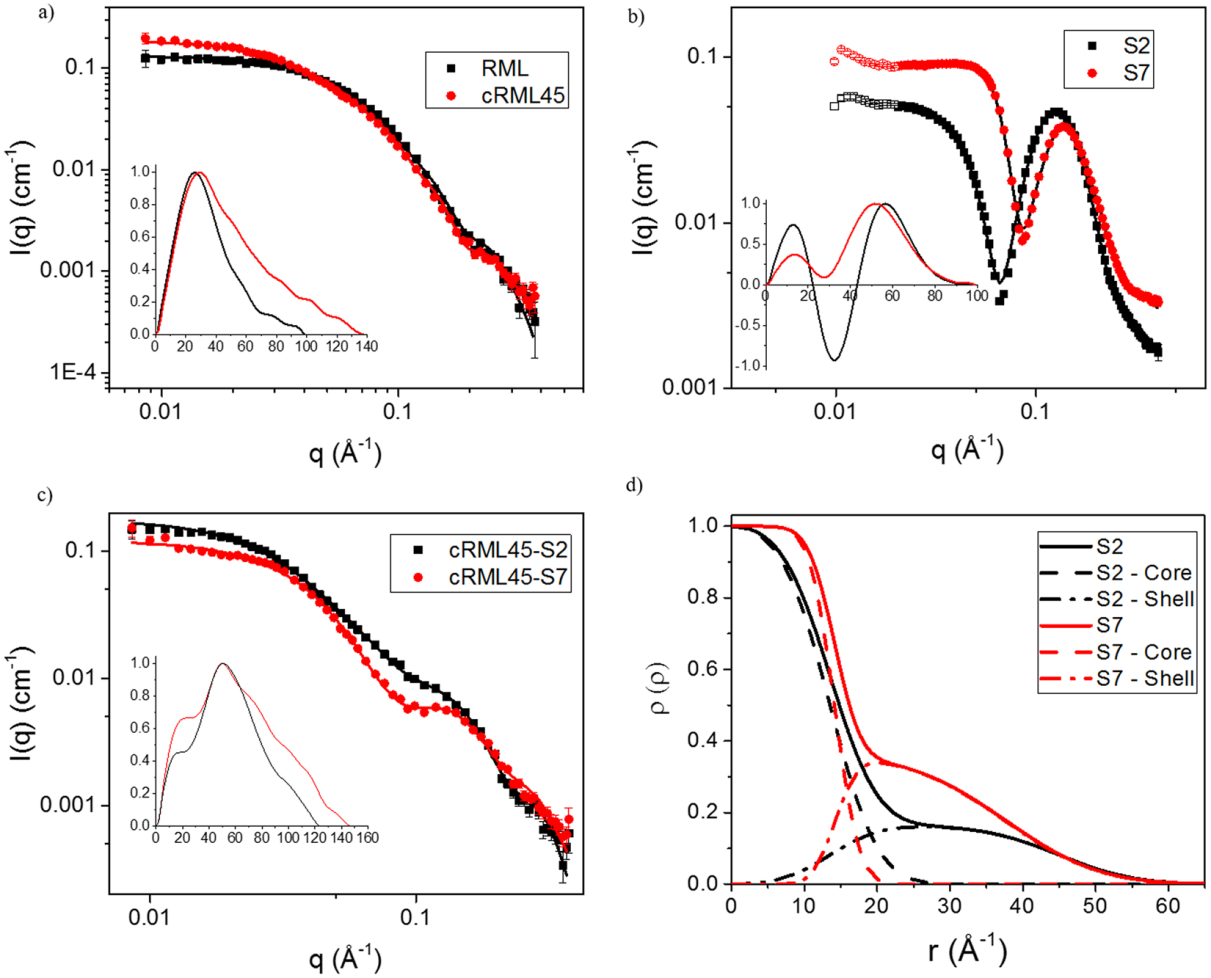
SAXS analysis of pure protein, surfactant and the resulting complexes. SAXS data with model fits for (**a**) RML and cRML45, (**b**) S2 and S7 and (**c**) cRML45-S2 and cRML45-S7. Insert in (**a**–**c**) are the corresponding p(r) functions determined from IFT as a function of q (Å-1). (**d**) Radial volume fraction profiles of S2 and S7 obtained from modelling in panel B. No buffer was used. RML analyzed was obtained after dialysis and freeze-drying.

SAXS is a biophysical method that provides low resolution information about the shape, conformation, and assembly state of various macromolecules^19^. Using the pair-distance distribution function (*p*(*r*)) to get model-independent real space information of protein, Fig. 4a showed that the shape of the RML was not that of globular monomeric protein. *I*(0) demonstrated that RML had a mass close to that of a dimer, while cRML45 had the mass of a trimer. A P2 dimer model was fitted to SAXS data for RML and a trimer model fitted to cRML45, showing good agreement at low and intermediate *q* on absolute scale (Fig. 4a). Before evaluating the biofluids which contain cationized enzyme and polymer surfactants, simpler systems containing exclusively polymer surfactants (S2 or S7) were studied for comparison purposes. S2 and S7 are both expected to form micelles above their critical micelle concentration. In fact, a core-shell spherical model with a structure factor accounting for inter-micelle interaction could fit the SAXS data (Fig. 4b). This core-shell structure is also evident from the *p(r)* function that has two characteristic maxima and a minimum (Fig. 4b). The minimum seen in both the SAXS data and the *p(r)* function arise from the opposite signs of the excess electron densities of the shell and core. On absolute scale, the modelling gave an aggregation number of 37 for both S2 and S7 in good agreement with an aggregation number of 40 for Brij35^20^, which, however, does not have the carboxylic acid at the end of the PEG chain. The radial volume fraction profiles are shown in Fig. 4d. The core is less sharp for the S2 micelles than for S7. Surprisingly, the width of the shell is almost the same, with a higher volume fraction for S7 in order to accommodate the larger volume of the chains for this surfactant. The similar width of the shell means that the PEG chains of S2 are more stretched than those of S7. Fig. S4 shows 3D representative structures of the micelles.

Both cRML45-S2 and cRML45-S7 produced SAXS data with a characteristic “bump” at high q values, similar to what was seen for the core-shell micelles even though less pronounced (Fig. 4c). The p(r) function (Fig. 4c) displayed a shoulder at intermediate distances (~2–3 nm) typical for the core-shell structure. The change of the location of the shoulder from 2.1 nm for cRML45-S7 to 2.8 nm for cRML45-S2 suggests that the core of the complex is bigger for cRML45-S2. Furthermore, the p(r) function also gives information on the maximum distances in the histogram, which is bigger for cRML45-S2 (15 nm) than for cRML45-S7 (12 nm).

**Figure 5:**
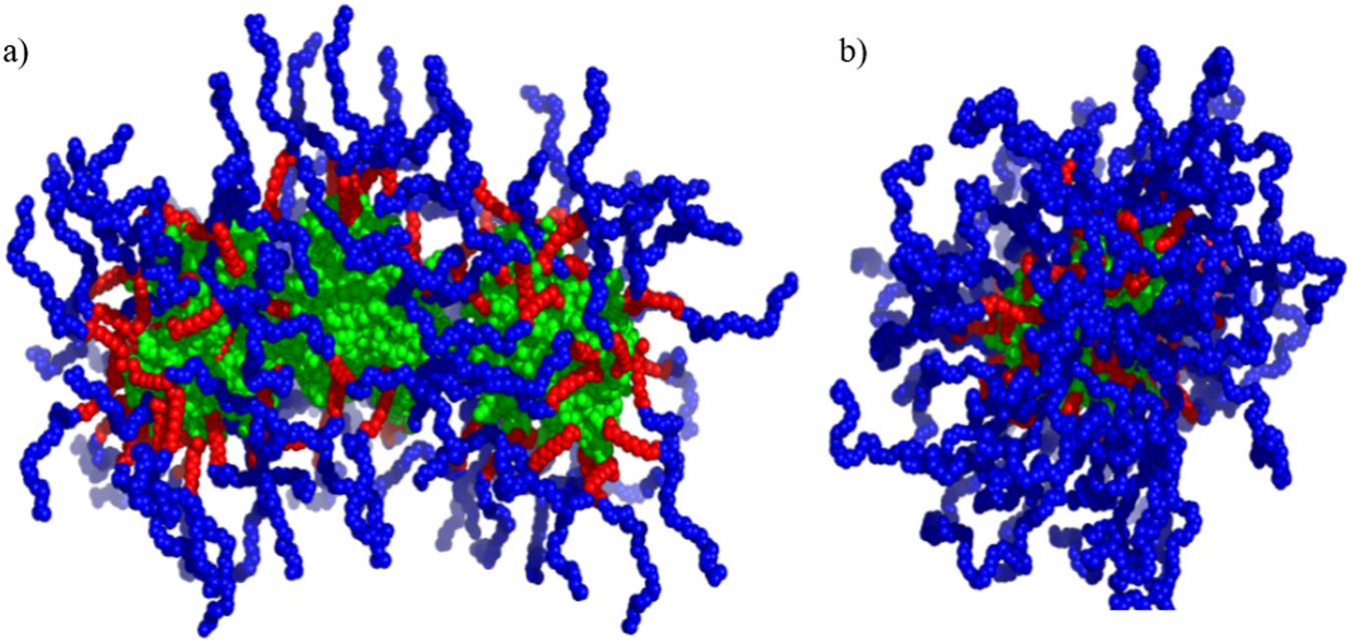
Representative 3D structures of cRML45-S2 (**a**) and cRML45-S7 (**b**). The protein, alkyl chains, and PEG chains are shown in green, red, and blue, respectively.

The data cannot be fitted by models with a core of surfactant and a protein corona without assuming a large degree of unfolding of the protein, which is not in agreement with the CD measurements as well as enzyme activity measurements that suggest that the protein is folded. Instead, we propose a model where the core consists of the folded protein surrounded by a shell of the surfactant. The models for the complexes must agree with the absolute scale of the SAXS data and with the known concentrations of protein and surfactant in the samples. This leads to a trimer model for cRML45 in the core for cRML45-S2 and a protein concentration of 1.9 ± 0.1 mg/ml compared to the measured 1.7 mg/ml (Fig. 5). For cRML45-S7 the data could be fitted with a core consisting of a single cRML45 giving a protein concentration of 1.1 ± 0.1 mg/ml compared to the measured 1.4 mg/ml (Fig. 5). This size difference of the core also explains the shifted shoulder in the p(r) function as well as the larger size of the cRML45-S2 complex (Fig. 4c). As mentioned previously, free micelles of the surfactants also had to be included in order to obtain good fits, and it should be noted that this also contributes to the core-shell signatures in the p(r) function. The optimized models gave complexes for cRML45-S2 with three proteins and 175 S2 molecules giving 35 surfactants per protein and 70 S2 molecules per protein in free micelles. The model for cRML45-S7 consists of one protein surrounded by 65 S7 molecules, while free micelles constituted 36 S7 per protein, which gives a total of 101 S7 molecules per protein in the sample. The number of polymer surfactants interacting with the enzyme could influence the activity of the enzyme by decreasing mass transfer^21,22^.

For cRML45-S7 the protein has a radius of 2.1 nm with a 0.3 nm thick shell of alkyl chains. cRML45-S2 has the 3 proteins in a prolate ellipsoidal shape of radius 2.3 nm and a length of 9.7 nm with a 0.4 nm thick shell of alkyl chains. The proposed models where the cationic lipase structure is confined by anionic polymer surfactants could explain the increased thermostability of the enzyme since aggregation of the enzyme could be prevented by less exposure of hydrophobic residues in the partially unfolded protein, which are buried in the native state^23,24^. Accordingly, the steric stabilization by the polymer surfactants in the biofluid enzymes combined with the electrostatic repulsion between the cationized enzymes could potentially prevent this self-association. Moreover, the SAXS predicted model could also explain the ability of the enzymes to recover their secondary structure after thermal stress conditions as the enzyme mobility is restricted by the polymer shell.

### Hydrolytic activity of the new biofluid lipases using *p*-nitrophenyl palmitate as substrate

**Figure 6:**
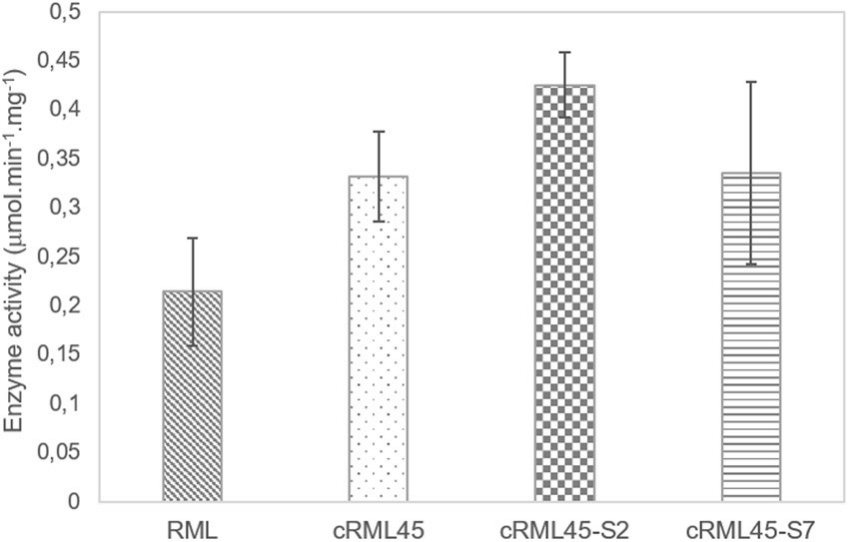
Hydrolytic activity of RML (obtained after dialysis and freeze-drying), cRML45, and new biofluid lipases using *p*-nitrophenyl palmitate as substrate in 50 mM of aqueous phosphate buffer at pH = 8 and room temperature. Enzyme concentration was measured by BCA assay (0.5 mg/mL).

The stability of the enzyme fold and function can be distinguished^23^. The former relates to the thermodynamics of the protein folding, while the latter describes the ability of the enzyme to remain catalytically active. Thus, it is of interest to also evaluate the influence of the length/number of PEG chain on the enzyme activity. Accordingly, the hydrolytic activity of RML (solid form), cRML45, cRML45-S2, and cRML45-S7, prepared for the purpose of this work, were measured using *p*-nitrophenyl palmitate as a substrate in 50 mM aqueous phosphate buffer at pH of 8 and room temperature. The enzyme activity of RML (solid form) after dialysis and freeze-drying was measured for comparison purposes since the solid enzyme was used to prepare the biofluids. However, solid RML displayed significantly lower activity (RML = 0.21 ± 0.05 μmol min^−1^ mg^−1^) than the liquid enzyme acquired from Sigma-Aldrich (≥20 U/mg). Regardless, as seen in Fig. 6 cRML45 (0.33 ± 0.05 μmol min^−1^ mg^−1^), cRML45-S2 (0.43 ± 0.03 μmol min^−1^ mg^−1^), cRML45-S7 (0.34 ± 0.09 μmol min^−1^ mg^−1^) displayed superior activity compared to solid RML (0.21 ± 0.05 μmol min^−1^ mg^−1^) demonstrating that cationization and/or polymer conjugation conferred additional stability to the enzyme. Compared to previous reports where a 60% of cationization efficiency was achieved for RML when the enzyme was modified at room temperature^7^, modifying RML at low temperature (<4 ^o^C) yielded a more active cationized form of the enzyme (cRML45 0.33 ± 0.05 μmol min^−1^ mg^−1^ *vs* cRML60 = 0.11 ± 0.04 μmol min^−1^ mg^−1^). The latter can be attributed to the low temperature (<4 °C) used to perform the cationization since it is hypothesized that the enzyme is less flexible at this temperature and buried residues (e.g. ASP of the catalytic triad) are less prone to be modified. Contrary, at room temperature, the enzyme is expected to be more flexible and the buried amino acids become exposed and are more likely to be cationized; thus the lower activity of cRML60 previously reported. In addition, when the cationized enzyme was made interact with the polymer surfactant S2 through non-covalent interactions a partial increase of the activity was observed (cRML45 0.33 ± 0.05 μmol min^−1^ mg^−1^ vs cRML45-S2 0.43 ± 0.03 μmol min^−1^ mg^−1^). This difference could be correlated with an increase of the α-helix content for cRML45-S2 compared to the CD results of cRML45. In addition, cRML45-S2 (0.43 ± 0.03 μmol min^−1^ mg^−1^) displayed higher activity than cRML45-S7 (0.34 ± 0.09 μmol min^−1^ mg^−1^). The lower activity observed for cRML45-S7 compared to cRML45-S2, could be a result of the number of polymers interacting with the protein (35 and 65 polymer surfactants for cRML45-S2 and cRML45-S7, respectively, as predicted by SAXS). Previous reports showed that increasing the number of PEG polymers bound to an enzyme can lead to lower enzyme activity^21,22^. Thus, though the number of S7 non-covalent bound to the protein seems to stabilize the cationized enzyme, they may hamper the interaction of the substrate with the catalytic triad of the enzyme decreasing mass transfer. However, it is possible that the length of the PEG chain may also be influencing substrate accessibility. Previous reports using L-asparaginase demonstrated that an increase on the number and length of PEG polymer decreases the enzyme activity^22,25^. Conversely, another report demonstrated that the enzyme activity of PEGylated trypsin did not depend on the length of the PEG polymer as 2 kDa, 5 kDa, and 10 kDa PEGylated trypsin displayed comparable activity to free trypsin and only the 20 kDa PEGylated trypsin showed lower activity than all other trypsin variants studied^17^. Regardless, it can be concluded that cRML45, was successfully modified by conjugation of S2 and S7 because the success of any protein-polymer conjugation approach is dependent on the ability of the protein to retain its activity after modification^26^. Previous reports using cRML60 demonstrated that the activity of the cationized enzyme dropped significantly after polymer conjugation (cRML60 0.11 ± 0.04 μmol min^−1^ mg^−1^ vs cRML60-S7 0.05 ± 0.01 μmol min^−1^ mg^−1^). To our knowledge, no previous report exists of a biofluid enzyme with activity comparable/superior to its cationized counterpart.

### Molecular dynamics simulations of biofluid lipases

MD simulations were performed to study the molecular interactions of the non-covalent complex between the anionic polymer surfactants and the cationized protein. Accordingly, previous reported biofluids lipase (cRML with cationization efficiency of ~60%; cRML60) were used to design the 3D-structures^7^. Although the modification of RML yielded different cationized version of RML, the major product of the cationization was cRML60, thus, to simplify the nature of the system, only cRML60 was used for this study. MD simulations were performed for RML, cRML60, cRML60-S7 at different temperatures (298 K, 348 K, 368 K) in aqueous medium for 500 ns. In addition, to evaluate the influence of the length of the PEG chain on the enzyme accessibility, cRML60-S2 was also generated and studied.

The monomer of RML presents a globular structure with Asp, Glu, Lys and Arg well distributed on the surface of the enzyme^27^. The catalytic triad consists of Ser144, Asp203, and His257. This enzyme possesses a lid (residues 82 to 96) that enables substrate selectivity. The 3D structure of cRML60 was built following a similar procedure previously reported^14^. Initially, 60% of Asp and Glu of the protein were identified as solvent exposed residues by propka.org (http://nbcr-222.ucsd.edu/pdb2pqr_2.0.0/) and cationized. Subsequently, multiple units of the surfactant were docked on the surface of the enzyme using BUDE^28^. Then the lipases and biofluid lipases were respectively equilibrated in aqueous conditions. To decrease the size of the system, only the polymers required to neutralize the cationized enzyme were included. Regardless, the concentrations of the polymer surfactants in the resulting systems were above the CMC of S2 (91  μM; Sigma-Aldrich) and S7 (>0.1 mM)^29^ as in the experimental set up. MD simulations of 500 ns were performed in triplicates using the AMBER99SB-ILDN force field^30^. After 12 ns of simulation, the polymer surfactants (S2 and S7, respectively) distributed at different points of the surface of the enzyme and some polymers agglomerated to form two hemi-micelles-like structures. In these micelle-structures, the hydrophobic tail of the surfactant generally pointed inwards. Similar results were displayed by previous simulated structures of biofluid myoglobin and biofluid lysozyme^14^. Moreover, within 12 ns, one of the micelle-structures reached the entrance of the catalytic triad and remained there in both cRML60-S2 and cRML60-S7 simulations after 500 ns of MD simulation. Similar results were observed among the replicas. This finding may explain the reduction of enzyme activity previously observed when the cationized enzyme (cRML60; enzyme activity = 0.11 ± 0.04 μmol min^−1^ mg^−1^) was made interact with S7 (cRML60-S7; enzyme activity = 0.05 ± 0.01 μmol min^−1^ mg^−1^)^7^. Also, it might explain the reduce activity of cRML45-S7 (0.34 ± 0.09 μmol min^−1^ mg^−1^) compared to cRML45-S2 (0.43 ± 0.03 μmol min^−1^ mg^−1^) obtained in the present work as the position of the surfactants on the enzyme surface (near the entrance of the catalytic triad; Fig. S5) combined with the size of the polymer might hamper the access of the substrate to the catalytic triad. In addition, MD simulations showed that the interactions of the polymers with the enzyme confined the protein and limits its fluctuations as previously predicted by SAXS experiments.

**Figure 7:**
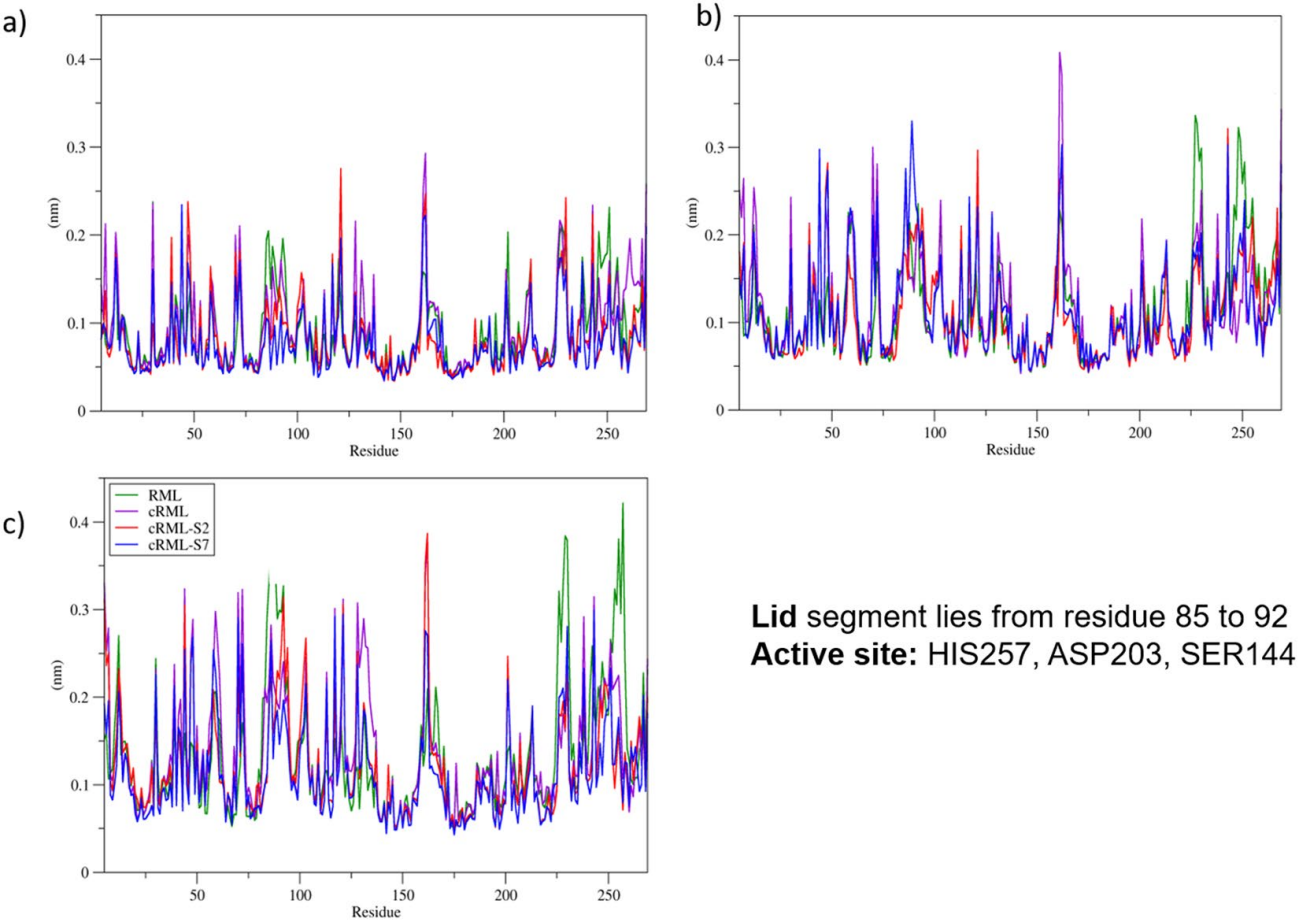
Root mean square fluctuation (RMSF) plots from the last 100 ns of 500 ns MD simulations of the different RML (Green), cRML60 (cationized RML where only 60% of ASP and GLU of the enzyme were modified; Purple), cRML60-S2 (Red), and cRML60-S7 (Blue). The MD simulations were done in aqueous conditions at 298 K, 348 K, 368 K.

To further characterize the protein-polymer interactions, different structural parameters were measured from the last 100 ns of the simulations. These structural properties were root mean square deviation (RMSD) and root mean square fluctuation (RMSF), radius of gyration, number of amino acid residues in the secondary structure, number of hydrogen bonds, solvent accessible surface area (SASA), and number of contacts. Results of the analysis of the MD simulations are summarized in Tables 2–4, and Fig. 7. In addition, RMSD results of the entire 500 ns MD simulations are included in the supplementary information (Figs S6–S9). As seen in Table 2, the RMSD increases as the temperature increases for all the lipases variants except for cRML60-S7, for which the RMSD did not change when increasing the temperature from 348 K to 368 K (RMSD = 0.21 ± 0.01 nm). For instance, the RMSD of RML increases from 0.15 ± 0.03 nm at 298 K to 0.26 ± 0.01 nm at 348 K and to 0.29 ± 0.03 nm at 368 K. These results agree with a decrease in the number of amino acid residues of cRML60-S2, cRML60, RML keeping their secondary structure. Interestingly, the number of residues in RML60-S7 retaining their secondary structure did not vary from 348 K (173 ± 3) to 368 K (174 ± 6). Furthermore, these data correlate with lower levels of fluctuations observed for cRML60-S7 compared to cRML60-S2, cRML60, and RML. Thus, the drop of enzyme activity for biofluids containing S7 polymer surfactant could be also explained by an increase of enzyme rigidity for this conjugate. Previous work using α-chymotrypsin reported that a decrease in enzyme activity upon PEGylation could be caused by an increase in enzyme rigidity^21^. In contrast with the results found for RML, cRML60, and cRML60-S2, the number of hydrogen bonds for cRML60-S7 increased from 201 ± 2 at 348 K to 207 ± 2 at 368 K. MD simulations results are in agreement with CD spectroscopy results which suggested that cRML45-S7 is more thermostable than RML, cRML45, cRML45-S2. No significant variations were observed for the radius of gyration when increasing the temperature. The lower SASA obtained for RML compared to the modified counterparts, can be explained by the elongated side chain present in the cationized ASP and GLU of cRML60.

**Table 2:** Structural features of the different lipases variants in aqueous conditions.

Enzyme	T ^a^(K)	RMSD (nm)	Radius of Gyration (nm)	Secondary Structure (# of Residues)	SASA (nm)	
					SC^b^	Main chain + H
RML	298	0.15 ± 0.03	1.71 ± 0.01	176 ± 5	100 ± 1	16.6 ± 0.2
	348	0.26 ± 0.01	1.71 ± 0.01	172 ± 4	100 ± 1	17.0 ± 0.1
	368	0.29 ± 0.03	1.72 ± 0.01	167 ± 3	100 ± 1	17.3 ± 0.1
cRML60	298	0.14 ± 0.02	1.70 ± 0.01	182 ± 8	121 ± 7	14.5 ± 2.2
	348	0.22 ± 0.01	1.71 ± 0.01	172 ± 3	121 ± 6	15.2 ± 2.3
	368	0.27 ± 0.03	1.72 ± 0.01	170 ± 2	122 ± 7	15.2 ± 2.3
cRML60-S2	298	0.15 ± 0.02	1.72 ± 0.01	176 ± 2	121 ± 4	14.4 ± 0.7
	348	0.20 ± 0.02	1.73 ± 0.02	174 ± 7	126 ± 4	15.1 ± 1.1
	368	0.25 ± 0.03	1.74 ± 0.01	169 ± 2	128 ± 2	15.9 ± 0.6
cRML60-S7	298	0.12 ± 0.01	1.70 ± 0.01	183 ± 1	123 ± 1	14.0 ± 0.5
	348	0.21 ± 0.01	1.72 ± 0.01	173 ± 3	126 ± 1	15.3 ± 1.4
	368	0.21 ± 0.01	1.72 ± 0.01	174 ± 6	126 ± 3	15.5 ± 2.8

The results presented are the average of three replicates from the last 100 ns of 500 ns of MD simulations.

^a^T = Temperature; ^b^SC = side chain.

**Table 3:** The number of intramolecular hydrogen bonds for the different lipase variants and the number of hydrogen bonds interaction between the polymer surfactants and cationized enzyme in aqueous conditions.

Enzyme	T (K)^a^	# of Hbond Enzyme/Enzyme^b^	# of Hbond Enzyme/S2(S7)			
			Head^c^	Body^d^	Normalized by # of molecules	
					Head	Body
RML	298	218 ± 4	—	—	—	—
	348	217 ± 3	—	—	—	—
	368	212 ± 3	—	—	—	—
cRML60	298	205 ± 2	—	—	—	—
	348	207 ± 5	—	—	—	—
	368	201 ± 9	—	—	—	—
cRML60-S2 (27 × S2)	298	211 ± 3	34.6 ± 3.3	13.9 ± 2.1	1.3 ± 0.1	0.5 ± 0.1
	348	204 ± 8	36.0 ± 3.0	9.9 ± 1.6	1.3 ± 0.1	0.4 ± 0.1
	368	200 ± 3	37.6 ± 5.4	12.9 ± 0.4	1.4 ± 0.2	0.5 ± 0.1
cRML60-S7 (24 × S7)	298	214 ± 2	20.6 ± 3.7	24.5 ± 4.3	0.9 ± 0.2	1.0 ± 0.2
	348	201 ± 2	20.9 ± 2.0	24.7 ± 4.3	0.9 ± 0.1	1.0 ± 0.2
	368	207 ± 2	22.5 ± 0.4	23.1 ± 1.8	0.9 ± 0.1	1.0 ± 0.1

The results presented are the average of three replicates for the last 100 ns of 500 ns of MD simulations.

^a^T = Temperature; ^b^Number of intramolecular hydrogen bonds of the enzyme; ^c^Head = COO^−^;

^d^Body = (-OCH_2_CH_2_-)_n_.

**Table 4:** Number of contact of the polymer surfactants with the enzyme in aqueous conditions.

Enzyme	T (K)	# of contacts					
		Head^a^		Body^b^		Tail^c^	
		BB^d^	SC^e^	BB	SC	BB	SC
cRML60-S2 (27 × S2)	298	14.1 ± 4.8	176 ± 1.6	123 ± 2.0	728 ± 8.9	34.4 ± 1.4	255 ± 7.4
	348	20.9 ± 3.0	178 ± 1.2	119 ± 1.1	703 ± 4.9	20.1 ± 5.2	216 ± 5.2
	368	26.0 ± 11	175 ± 13	122 ± 8	746 ± 4	22.4 ± 3.5	211 ± 4
cRML60-S7 (24 × S7)	298	9.5 ± 3.9	133 ± 11	155 ± 15	887 ± 65	29.0 ± 8.1	238 ± 5
	348	12.0 ± 3.0	132 ± 4	151 ± 10	912 ± 9	17.3 ± 0.3	144 ± 2
	368	12.9 ± 1.1	133 ± 5	170 ± 27	941 ± 109	18.3 ± 2.6	171 ± 18

The results presented are the average of three replicates from the last 100 ns of 500 ns of MD simulations.

^a^Head = -COO^−^; Body = (-OCH_2_CH_2_-)_n_; ^c^Tail = CH_3_(CH_2_)_11_; ^d^BB = Backbone; ^e^SC = side chain.

In addition, the number of contacts and the number of hydrogen bonds of the polymer surfactants (head = carboxylate, body = PEG, and tail = hydrophobic chain) with the enzyme were calculated. As seen in Tables 3 and 4, the head of the polymer S2 forms more hydrogen bonds and has a higher number of contacts with the protein than S7. Moreover, as expected, the body of S7 displayed increased number of contacts and hydrogen bond interactions compared to S2 and the increase correlates with the number of ethylene glycol units of the polymers. For example, S2 and S7 displayed 0.5 ± 0.1 and 1.0 ± 0.2 hydrogen bonds, between the body of the polymer and the protein at 298 K, respectively. These findings are in agreement with lower RMSF observed for S7 compared to S2 complexes and might indicate the relevance of the number of ethylene glycol units in stabilizing the cationized enzymes (Fig. 7). Also, they showed that the carboxylate head does not contribute to this stabilization. However, it promotes the interaction of the polymer with the cationized sites of the enzyme. Thus, this data provides insight in the mechanism of PEG mediated stabilization of protein structure.

## Conclusions

FTIR spectroscopy, DSC and MALDI-TOF MS analysis and hydrolytic activity assays results of the different lipase variants demonstrated that S2 and S7 were successfully non-covalently grafted to the cationized enzyme to yield new biofluid lipases. Molecular dynamic simulation and experimental results suggest that S7 yields a more thermostable biofluid lipase form than S2, and experimental results suggest the latter yields a more active form of enzyme. Thus, the size, number and location of the polymer on the surface of the enzyme could influence both the enzyme activity and thermostability as the hydrogen network formed between the protein and the PEG-based polymer confers steric stabilization and the steric hindrance of the PEG-polymer may affect the mass transfer. However, systematically changing the position of the polymer surfactants to confirm that the location of the polymer influences the enzyme activity is not trivial in the current system for two reasons. Firstly, the enzyme is chemically cationized, which gives rise to a distribution of supercationic enzyme molecules and hence a distribution of enzyme-polymer surfactant conjugates. Secondly, as the bonding between the enzyme and the polymer surfactant is electrostatic, there is the possibility of headgroup “hopping”, as these bonds are labile when compared with the covalent attachment of PEG (PEGylation). However, in general, the number of sites that can be PEGyleted on a protein is generally lower than the current method (35 and 65 chains per enzyme for S2 and S7, respectively), and for this reason larger PEG molecular weights are often used. Accordingly, rationally changing the cationic charge distribution using mutagenesis to a level^31^ that is commensurate with the chemical cationization would almost certainly lead to problems in protein expression and purification, and are beyond the scope of this current study.

Moreover, results demonstrated that the cationization efficiency also plays a role in determining enzyme activity. Finally, SAXS data demonstrated that both cRML45-S2 and cRML45-S7 present a characteristic “bump” at high *q* values, similar to what it was seen for the core-shell micelles of S2 and S7 even though less pronounced. Based on the SAXS results, a 3D-model is presented where the core consists of the folded protein surrounded by a shell of the surfactant. This model is consistent with previous hypotheses introduced for biofluid enzymes^7,14^. In addition, these models might explain the ability of the enzyme to recover its secondary structure as lipase mobility seems to be restricted by a polymer shell. Together, these results provide insight into the mechanism of non-covalent PEG mediated protein stabilization and contribute to helping exploit the full potential of the biofluid enzyme for the development of new enzyme technology.

## Experimental

All chemical reagents were purchased from Sigma Aldrich. Liquid lipase from *Rhizomucor miehei* was also acquired from Sigma-Aldrich (Catalog number: L4277; ≥20 U/mg). The liquid enzyme was dialyzed against Milli-Q water for 48 h, and freeze-dried for 48 h to yield a white/transparent solid. This solid enzyme was used to prepare the biofluids and as a control in the different analysis performed in this work. The PDB structure 4TGL^27^ was used to carry out both *in silico* and SAXS analysis.

### Experimental studies

#### Synthesis of biofluid lipases

The biofluid forms of RML were synthesized following a similar procedure previously reported^7,10^. Initially, 13.2 mmol of DMPA (1 eq combined ASP and GLU to 300 eq of DMPA) were dissolved in 10 mL of water and the pH of the solution was adjusted to 6. The total volume of the DMPA solution was then increased to 50 mL and 50 mg of native RML (solid powder) were added. Then the pH was re-adjusted to 6 yielding a final enzyme concentration of approximately 1 mg/mL. The reaction vessel was subsequently cooled down <4 °C and the coupling reaction was initiated by the addition of 2.2 mmol EDC and 2.2 mmol of NHS. The coupling reaction was performed over night at low temperature (<4 °C) and the resulting solution was dialyzed against milli-Q water, filtered through a 0.45 μm filter, and freeze-dried. To yield the respective biofluid lipase, an aqueous solution at a pH 6.8 containing 0.028 mmol of polymer surfactant (S2 or S7) and 9–10 mL of Milli-Q water was prepared. Posteriorly, 10 mg of cationized RML (cRML45) were added to the surfactant solution and the pH was readjusted to 6.8 and the reaction was let run overnight. The resulting solution was dialyzed against Milli-Q water for 24 h and freeze-dried for 48 h (Buch and holm Alpha 1–2 LDplus; Denmark).

#### Matrix-Assisted laser desorption/ionization-time-of-flight mass spectrometry (MALDI-TOF MS)

The cationized and native RML were analyzed by MALDI-TOF MS following a procedure previously reported^32^. Briefly, an aliquot (1–5 μl) of the derivatized protein was lyophilized, resuspended in 2 μl 1% trifluoroacetic acid and mixed with 2 μl 2,5-dihydroxyacetophenone (0.1 M in 20 mM ammonium dihydrogen citrate and 75% (v/v) EtOH) [PMID: 16456805^33^]. 1 μl of the mixture was spotted onto a stainless steel target and allowed to dry. The spectra were recorded in positive and linear mode using an AutoFlex Smartbeam III instrument (Bruker) calibrated by external calibration (Peptide calibration standard I; Bruker Daltronics). Data were collected in the range of *m/z* 20,000–45,000. The data were processed by the flexAnalysis software (v. 3.3) and the centroid masses evaluated using the GPMAW software (gpmaw.com).

#### Circular Dichroism spectroscopy

The structure of the different lipase-polymer surfactant nanoconjugates was monitored by CD spectroscopy in a JASCO J-810 spectropolarimeter (Japan) fitted with Jasco Peltier-type temperature controller (PTC-423S; Japan). The concentration of the protein used was approximately 0.133 mg/mL in Milli-Q water. The cuvette had a path length of 0.1 cm. CD spectra between 190 nm and 250 nm were recorded at room temperature. The secondary structure content of each of the enzymes was calculated using Dichroweb web server^15,16^. Temperature dependent unfolding profiles were determined following changes at 222 nm while heating the solution at a constant rate of 5 °C per minute from 25 °C to 95 °C. Possible thermal refolding was monitored by scanning from 95 °C to 25 °C with the same rate. Full wavelength spectra (from 190 nm to 250 nm) for cRML45-S2 and cRML45-S7 were measured before and after heating.

#### SAXS measurements and initial data treatment

The flux- and background-optimized SAXS instruments at Aarhus University were used for collecting the data. One instrument is a prototype of the NanoSTAR SAXS camera from Bruker AXS and uses a Cu rotating anode x-ray source with a wavelength of λ = 1.54 Å. The other is a new NanoSTAR SAXS from Bruker AXS^34^ with a liquid metal Ga jet source (Excillum) with a wavelength λ = 1.34 Å and was used for the pure surfactant samples. Both instruments use a homebuilt scatterless pinhole^35^ in front of the sample. Solutions of 3.1 mg/ml RML, 2.9 mg/ml cRML45, 60 mg/ml S7 and 71 mg/ml S2 dissolved in water were used for the simple protein and surfactant systems. For the complexes, samples of 1.7 mg/ml cRML45 and 8.3 mg/ml S2 (1:205 cRML:S2 molar ratio), and 1.4 mg/ml cRML45 and 8.6 mg/ml S7 (1:146 cRML45:S7 molar ratio) dissolved in water were used. Data were recorded for 15 min for surfactant samples and 30 min for the rest. Measurements were performed at 20 °C and water was used for background subtraction. The SUPERSAXS program package (Oliveira, C.L.P. and Pedersen J.S, unpublished) was used for initial data treatment and conversion to absolute scale using water as a calibration standard. Intensities are plotted as a function of the modulus of the scattering vector, *q* = 4πsinθ/λ, where θ is half the scattering angle. Model-independent information was obtained by determining the pair-distance distribution functions, *p*(*r*), that gives information on real-space distances on the structures. The *p*(*r*)) function is a histogram of distances between pair of points weighted by the excess electron density at the points. The Indirect Fourier Transformation procedure^36^ was used for determining the *p*(*r*) function using the home-written program WIFT^37,38^.

The scattering from the crystal structures of RML was calculated together with that of the dimer and trimer to determine the structure of RML and cRML45 in water. In-house developed software was used for calculating the solution scattering from the RML PDB structure. The program uses an average atom for non-hydrogen atoms with average fixed mass, average number of electrons, and average volume corresponding to an excess scattering length per mass of 2.0 × 10^10^ cm/g (J.S. Pedersen and C.L.P. Oliveira, Unpublished data). The scattering is calculated using the Debye equation^39^ on absolute scale so that the protein concentration and a background are fit parameters, optimized by a standard least-squares routine. A hydration layer is generated by a fast routine which selects points placed on a grid and the excess scattering of this can be optimized. The PDB structure of the single RML molecule did not fit the SAXS data for RML and cRML45, and therefore oligomeric structures were generated by a random search algorithm (also home-written software), which includes non-overlap of the structures and contact of the molecules as constraints. A dimer with P2 symmetry could fit the data for RML, whereas a trimer without any symmetry could fit the cRML45 data. The fits somewhat deviated at large *q* (scattering vector) suggesting additional scattering possibly from glycosylation and disordered regions. This was included in the final fits by adding the scattering from Gaussian chains^40^.

The SAXS data from the micelles of S2 and S7 were modelled with a core-shell spherically symmetric structure, including a polymer contribution for describing the scattering from the PEG chains in the corona shell^41^. The model has graded interfaces between core and shell, and between shell and solvent. The modelling was done on absolute scale using electron densities calculated from the values given in the references^42,43^. In this calculation, the scattering of the carboxylic acid was taken as the same as that of PEG. An effective hard-sphere structure factor^44^ was implemented to account for the inter-micelle interactions influencing the SAXS data at the relatively high concentration used for these samples. The radial volume fraction profiles of the micelles were calculated using a numerical Fourier transform of the scattering amplitudes, as previously reported^41^.

Complexes of RML and surfactants were modelled as core-shell structures also on absolute scale. Since the proteins have enzyme activity, the proteins were modelled by the structure of the native protein. It was assumed that the protein is in the core surrounded by surfactant molecules attached to the surface of the protein. The size of the ellipsoid (or sphere) in the center of the model, describing a trimer (for cRML45-S2) or a monomer (for cRML45-S7) of the protein, was determined by fitting, respectively, an ellipsoid of revolution and a sphere to the scattering calculated for a monomer model and the scattering of the trimer of pure cRML45. The central ellipsoid/sphere was kept constant in the fit to the data from the complexes. In the model, the core is surrounded by a (thin) shell containing the alkyl chain of the surfactant and one more shell with a graded outer surface describing the PEG chains of the surfactant. Additional free micelles in the solution had to be included to obtain reasonable fits. The scattering of these was fixed at structures of the pure micelles except for the omission of the structure factor since the concentrations were much lower in the samples with the complexes.

#### Hydrolysis of para-nitrophenyl palmitate

Lipase activity was evaluated by monitoring the hydrolysis of *p*-nitrophenyl palmitate as previously described^45,46^. Briefly, first 207 mg of sodium deoxycholate, 100 mg of gum Arabic and 50 μL of triton 100-X were dissolved in 90 mL of aqueous phosphate buffer (50 mM) at pH 8 (Solution A). Second, 30 mg of *p*-nitrophenyl palmitate were dissolved in 10 mL of isopropanol/acetonitrile 9:1 ratio (Solution B). Then Solution A and Solution B were mixed to yield the substrate solution. For evaluating the enzyme activity, 480 μL substrate solution was mixed with 20 μL of enzyme solution (0.5 mg/mL of enzyme) and left to react for 15 min at room temperature. The formation of product was evaluated by measuring the absorbance at 401 nm using a UV-visible spectrophotometer (Cary 50Bio, Varian, Australia). The reactions were performed in triplicate.

### *In silico* studies

#### Enzyme preparation

The protein-polymer surfactant conjugates were built following a similar procedure previously reported^14^. Initially, the unmodified protein (PDB ID: 4TGL) was prepared using the protein preparation wizard included in the Schrödinger software release 2015–1^47,48^. The protonation state of residues at pH 7 was determined using propka^47^. Subsequently, ~60% of ASP and GLU of the protein (Residue index: 13, 39, 44, 47, 48, 70, 72, 113, 117, 121, 128, 161, 162, 201, 226, 230, 238 and 243) identified as solvent exposed residues by propka^47^ (http://nbcr-222.ucsd.edu/pdb2pqr_2.0.0/) were structurally modified to generate the cationized structure of the enzyme. The parameters of the new residues called ASD and GLD (ASP and GLU residues modified using DMPA previously established by Sessions’ research group using acpype and antechamber were added to AMBER99SB-ILDN forcefield^30,49^. The Force-field parameters of S2 likewise were acquired from Sessions’ group. The parameters of S7 were built following the same procedure as Sessions’ *et al*.^14^ used to generate the parameters for S2. The protein-polymer surfactant conjugates were constructed using the molecular docking program Bristol University Docking Engine (BUDE)^28^ to dock 10 copies of the polymer surfactant at 27 randomly selected points on the surface of the enzyme. The software GA_lig_picker; included in the BUDE program; was used to select the non-overlapping set of polymer surfactants.

#### Molecular dynamics simulations

The complex were solvated with GROMACS utilities editconf and gmx solvate using the TIP3P water model^50^. The solvent boxes (Dodecahedron) were 1.4 nm larger than the complexes. The boxes were relaxed by energy minimization, followed by a 10 ns simulation at 300 K, restraining the protein to the initial position. The polymers that dissociated from the enzyme surface were removed. Then, the systems were neutralized at pH = 7. The complexes were subsequently equilibrated at the temperature of interest (298 K, 348 K, and 368 K, respectively) for 1 ns with the full protein restrained, 1 ns with only the modified amino acid residue restrained and, finally, 10 ns using no-restrains, at a pressure of 1 bar. The resulting complexes were simulated for 500 ns using GROMACS 5.0.2. Three replicates were performed.

#### Molecular dynamics simulation analysis

The root mean square deviation, the number of amino acid residues involved in a secondary structure element, the radius of gyration, the hydrogen bond counts, the solvent accessible surface area, and the root mean square fluctuation were calculated using GROMACS utilities gmx rms, gmx do_dssp, gmx gyrate, gmx hbond, gmx sasa, and gmx rmsf, respectively. In addition, gmx mindist was used to calculate the number of contact of the polymer head, body and tail with the enzyme. VMD was used to visualize the structures and trajectories^51^.

### Data availability

The manuscript includes a synthetic procedure to yield active biofluid lipases, FTIR spectroscopy data, CD spectroscopy data, SAXS measurements, enzyme activity assays, and 500 ns MD simulations results at three different temperatures. Main results are summarized within 7 figures and 4 Tables inside the manuscript.

## Electronic supplementary material

Supplementary information
