# Supplementary material for: Insight into the molecular mechanism behind PEG-mediated stabilization of biofluid lipases
Source: Sci Rep. 2018 Aug 16;8:12293. doi: 10.1038/s41598-018-29871-z (PMC6095910; doi:10.1038/s41598-018-29871-z)
Supplement: Supplementary file 1 — Supplementary information [file 41598_2018_29871_MOESM1_ESM.docx]

**Supplementary information**

***Insight into the molecular mechanism behind PEG-mediated stabilization of biofluid lipases***

Bianca Pérez,^[1]*^ Andrea Coletta,^[2]^ Jannik N. Pedersen,^[2,3]^ Steen V. Petersen,^[4]^ Xavier Periole, ^[2]^ Jan Skov Pedersen,^[2,3]^ Richard B. Sessions,^[5]^ Zheng Guo,^[1]^ Adam Perriman,^[6]^ Birgit Schiøtt^[2,3]^

^[1]^Department of Engineering, Aarhus University, Aarhus 8000, Denmark;

^[2]^Department of Chemistry, Aarhus University, Aarhus 8000, Denmark;

^[3]^Interdisciplinary Nanoscience Center, Aarhus University, Aarhus 8000, Denmark.

^[4]^Department of Biomedicine, Aarhus University, Wilhelm Meyers Allé 4, Aarhus 8000, Denmark;

^[5]^School of Biochemistry, University of Bristol, Bristol BS8 1TD, U.K;

^[6]^School of Cellular and Molecular Medicine, University of Bristol, Bristol BS8 1TD, U.K.

*Corresponding author: Bianca Pérez, Ph.D.; Gustav Wieds vej 10C, Aarhus, DK-8000; email:bperez@eng.au.dk, phone: +4587155527.

Table of contents

[**Figure S1.** MALDI-TOF MS analysis of RML and cationized RML. 2](#_Toc509823948)

[**Figure S2.** FTIR spectra of the different lipases variants and polymer surfactants in their pure form (solid powder). 3](#_Toc509823949)

[**Figure S3.** CD spectra from 190 nm to 250 nm of the biofluid lipases in aqueous solution at room temperature (Blue) before temperature scan, at 95 ^o^C (Orange) after heating the sample from 25 °C to 95 °C, and at 25 °C (Gray) after cooling the sample from 95 °C to 25 °C. 4](#_Toc509823950)

[**Figure S4.** Representative 3D structures of the S2 (Left) and S7 (Right) micelles predicted based of the SAXS data for aqueous polymer solutions above the CMC. 4](#_Toc509823951)

[**Figure S5.** Representative structure of biofluid lipases after 500ns of MD simulations showing polymer surfactants agglomerating and forming a hemi-micelle like structures near the entrance of the catalytic pocket. 5](#_Toc509823952)

[**Figure S6.** Root mean square deviation (RMSD) plots from 500 ns MD simulations of RML in aqueous conditions at 298 K, 348 K, and 368 K, respectively. 6](#_Toc509823953)

[**Figure S7**. Root mean square deviation (RMSD) plots from 500 ns MD simulations of cRML60 in aqueous conditions at 298 K, 348 K, and 368 K, respectively 7](#_Toc509823954)

[**Figure S8.** Root mean square deviation (RMSD) plots from 500ns MD simulations of cRML60-S2 in aqueous conditions at 298 K, 348 K, and 368 K, respectively. 8](#_Toc509823955)

[**Figure S9.** Root mean square deviation (RMSD) plots from 500 ns MD simulations of cRML60-S7 in aqueous conditions at 298 K, 348 K, and 368 K, respectively 9](#_Toc509823956)

FIGURES

**
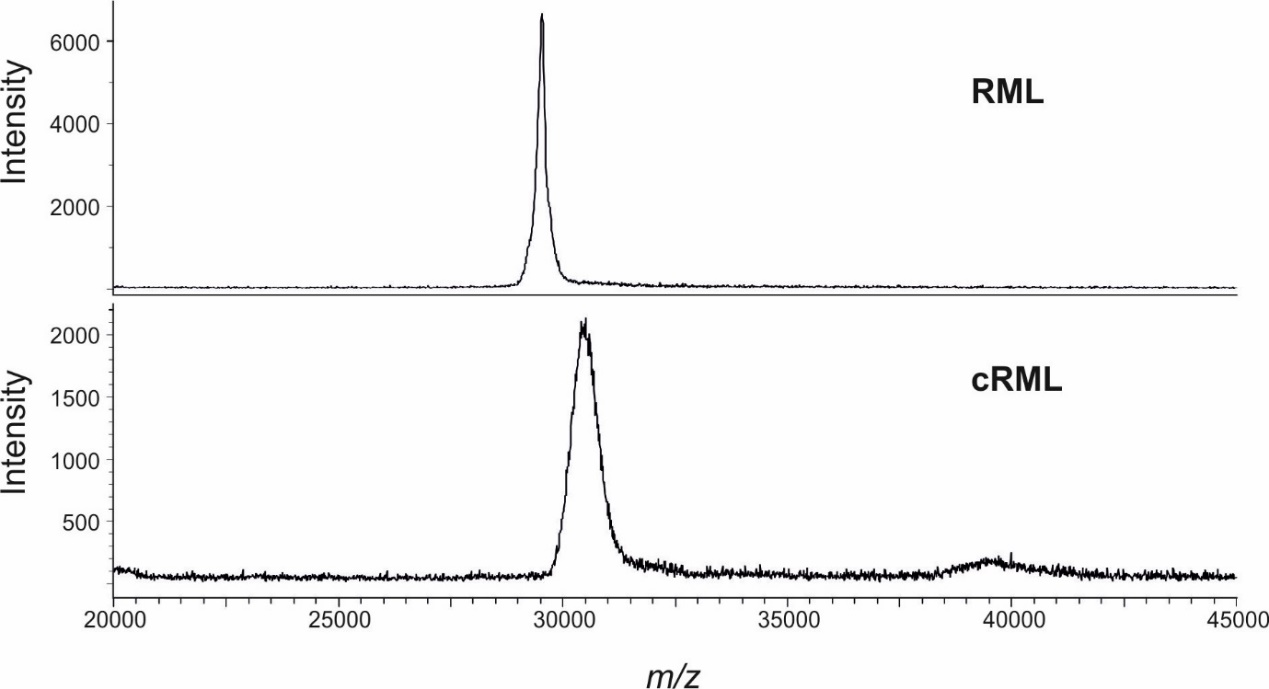
**

**45**

# **Figure S1.** MALDI-TOF MS analysis of RML and cationized RML.

# **Figure S2.** FTIR spectra of the different lipases variants and polymer surfactants in their pure form (solid powder).

# **Figure S3.** CD spectra from 190 nm to 250 nm of the biofluid lipases in aqueous solution at room temperature (Blue) before temperature scan, at 95 ^o^C (Orange) after heating the sample from 25 °C to 95 °C, and at 25 °C (Gray) after cooling the sample from 95 °C to 25 °C. Heating rate used was 5 °C per min.

**Figure S4.** Representative 3D structures of the S2 (Left) and S7 (Right) micelles predicted based of the SAXS data for aqueous polymer solutions above the CMC. The alkyl chains PEG chains are shown in red and blue, respectively.

**
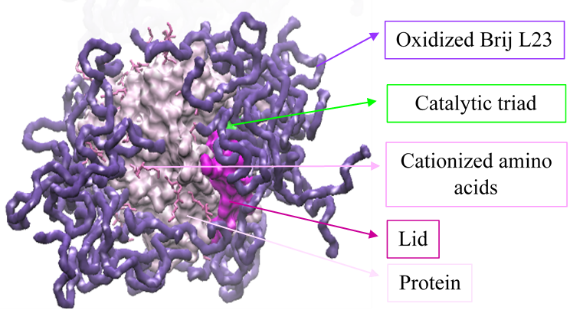
**

# **Figure S5.** Representative structure of biofluid lipases after 500ns of MD simulations showing polymer surfactants agglomerating and forming a hemi-micelle like structures near the entrance of the catalytic pocket.

**
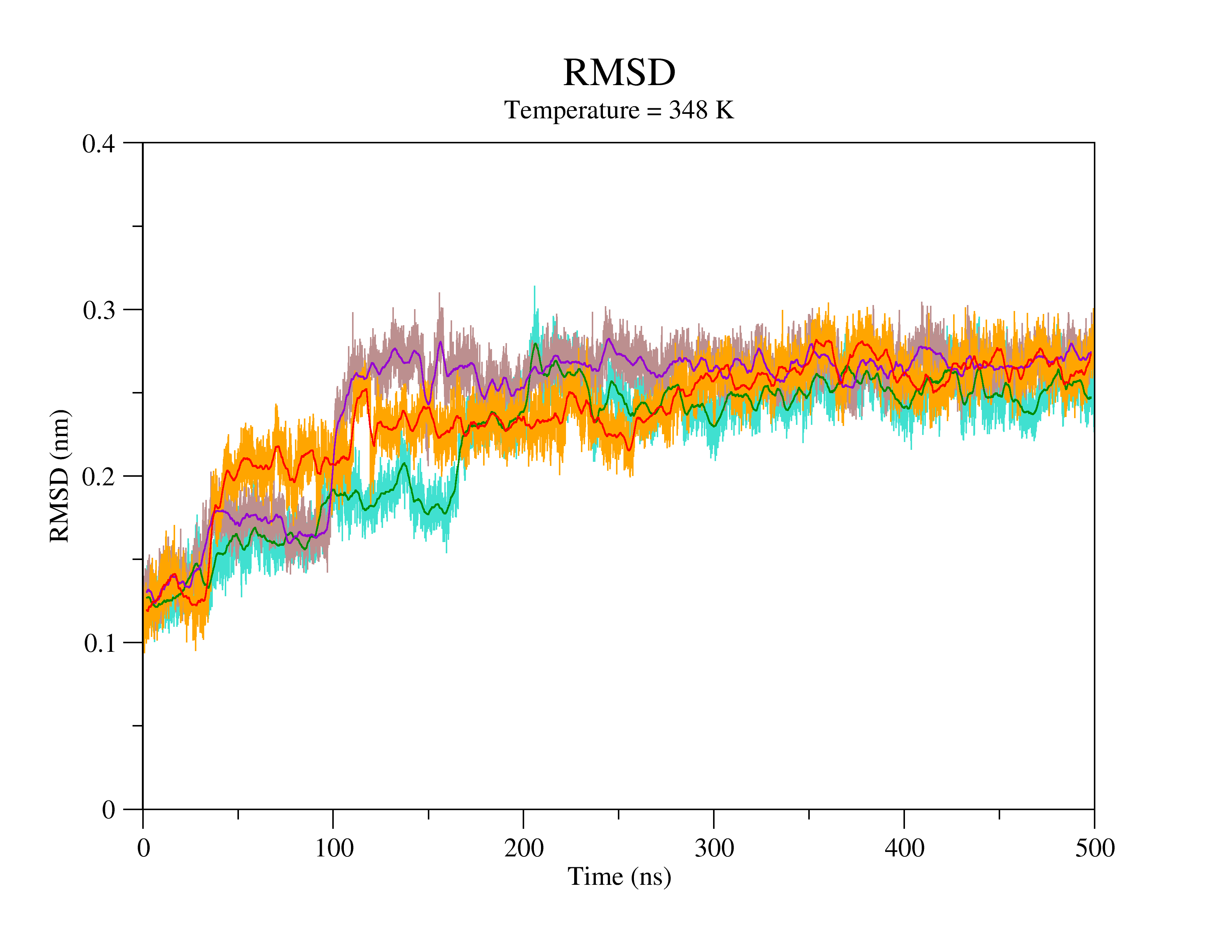
**


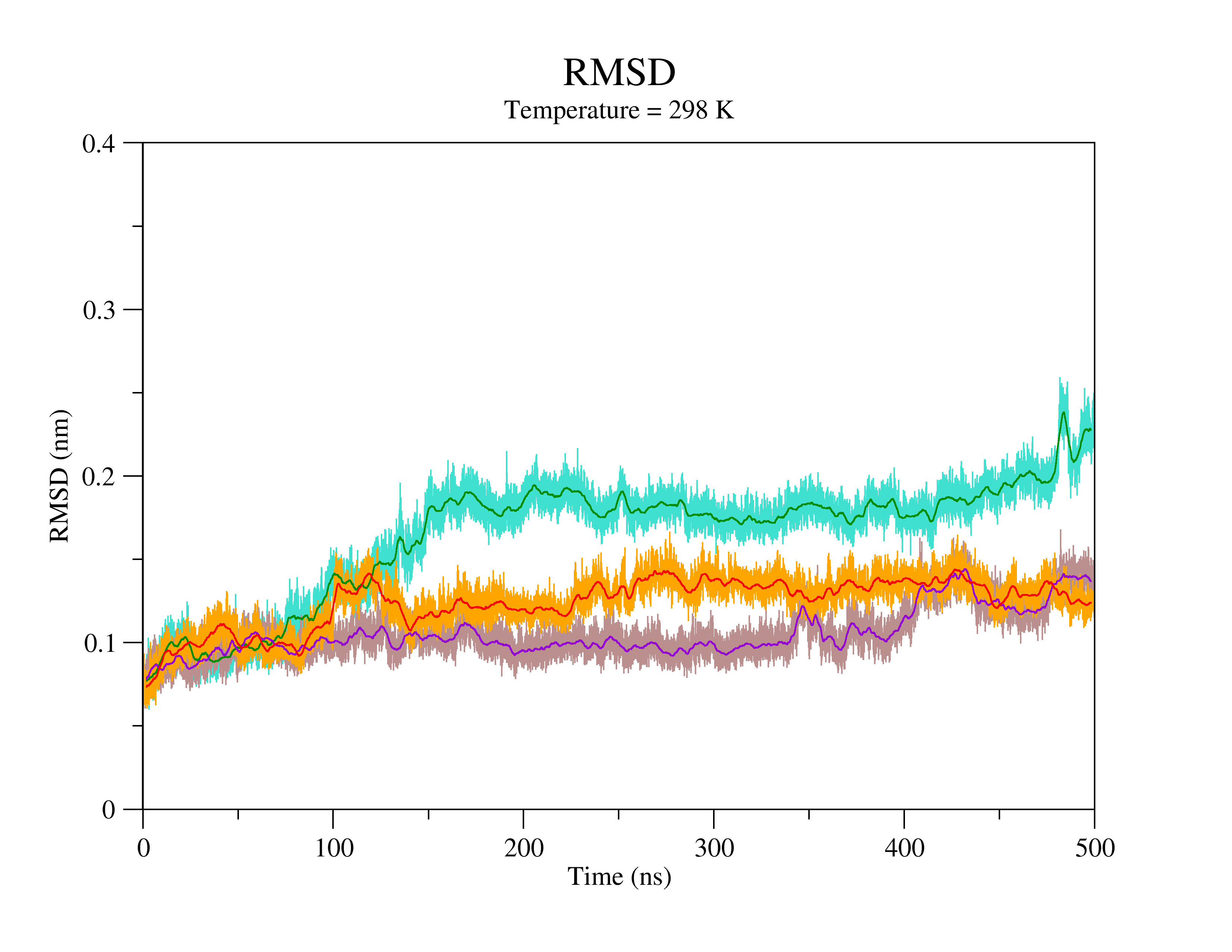

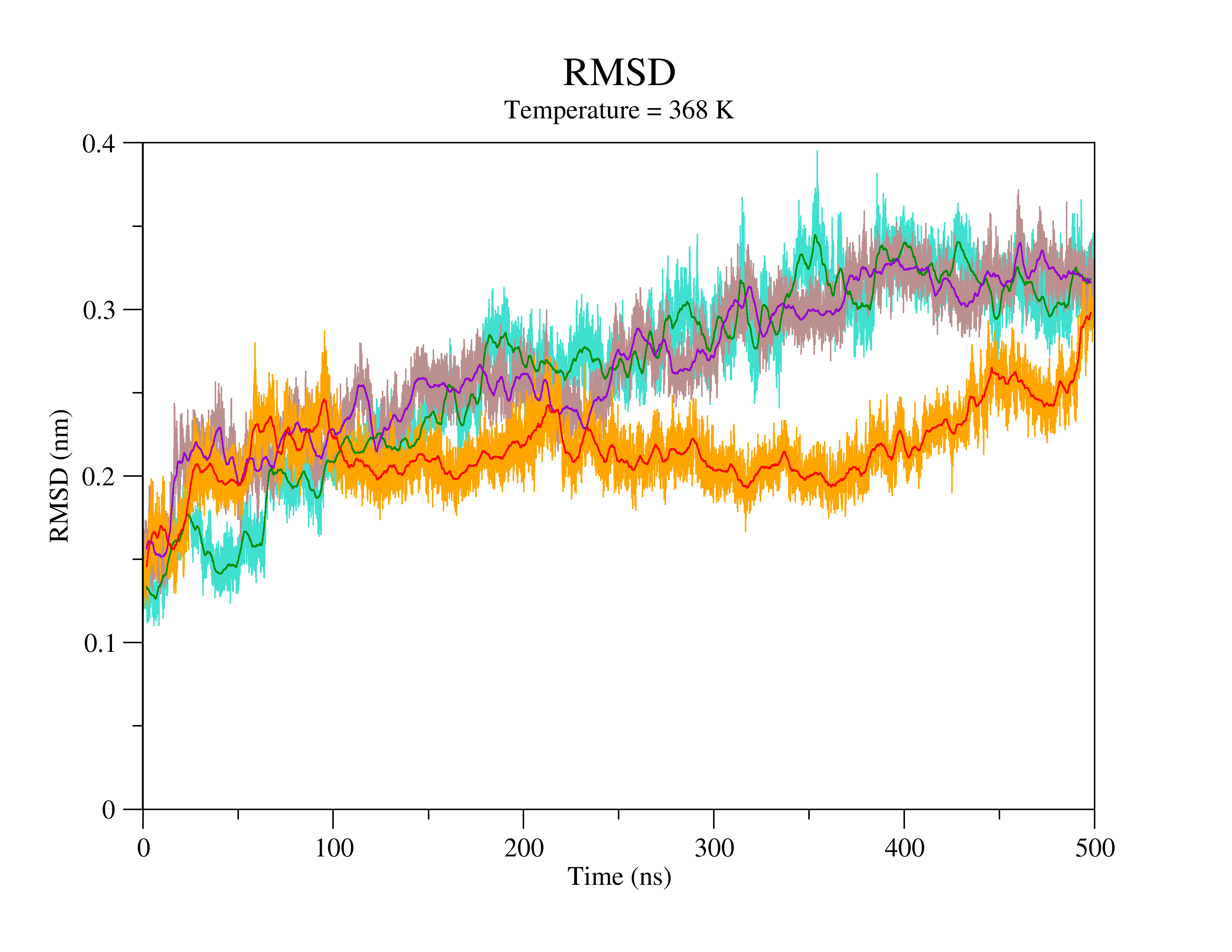


**Figure S6.** Root mean square deviation (RMSD) plots from 500 ns MD simulations of RML in aqueous conditions at 298 K, 348 K, and 368 K, respectively. Three replicates were done at each temperature. The running average of each of the replicates is also included.


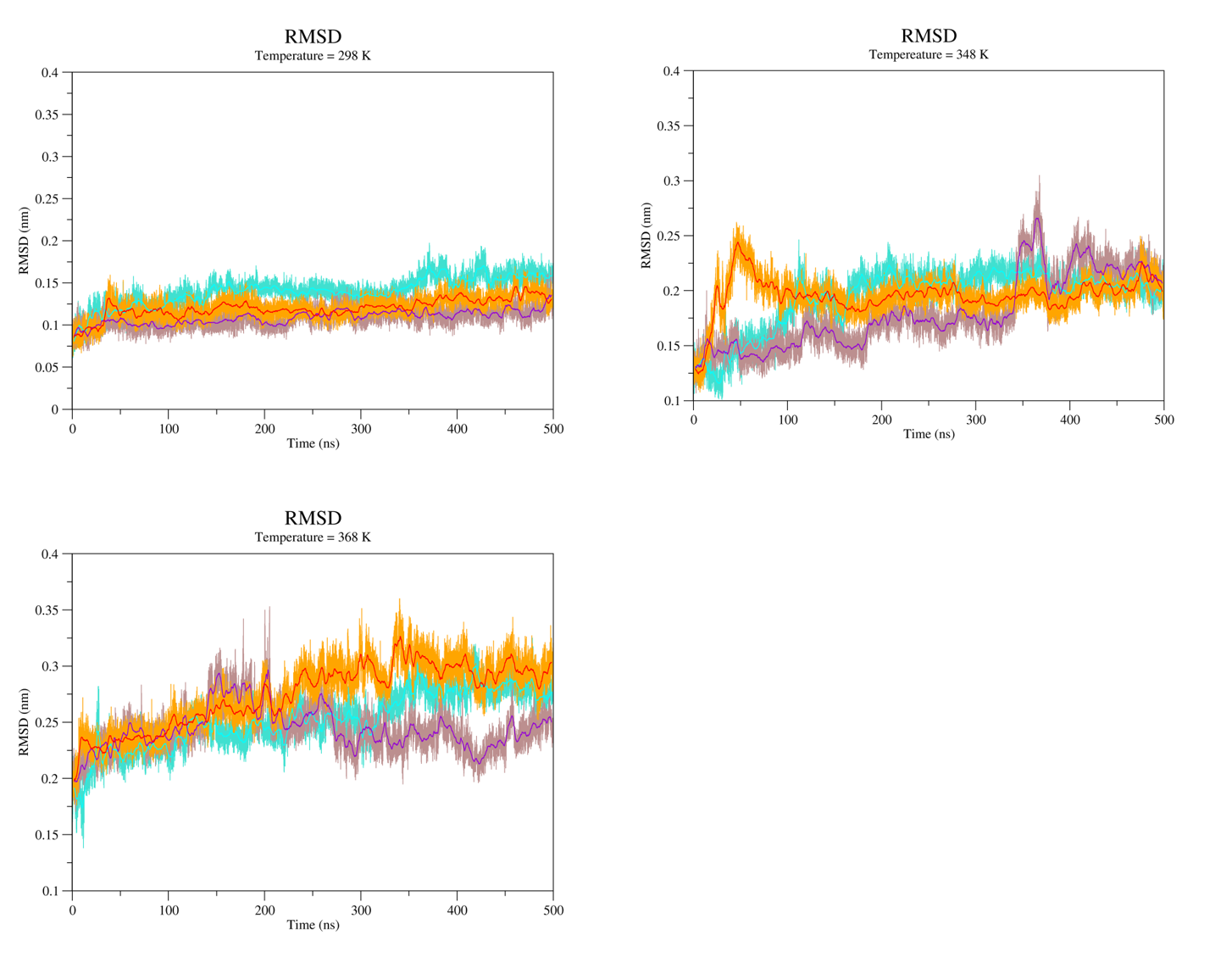


**Figure S7**. Root mean square deviation (RMSD) plots from 500 ns MD simulations of cRML60 in aqueous conditions at 298 K, 348 K, and 368 K, respectively. Three replicates were done at each temperature. The running average of each of the replicates is also included.


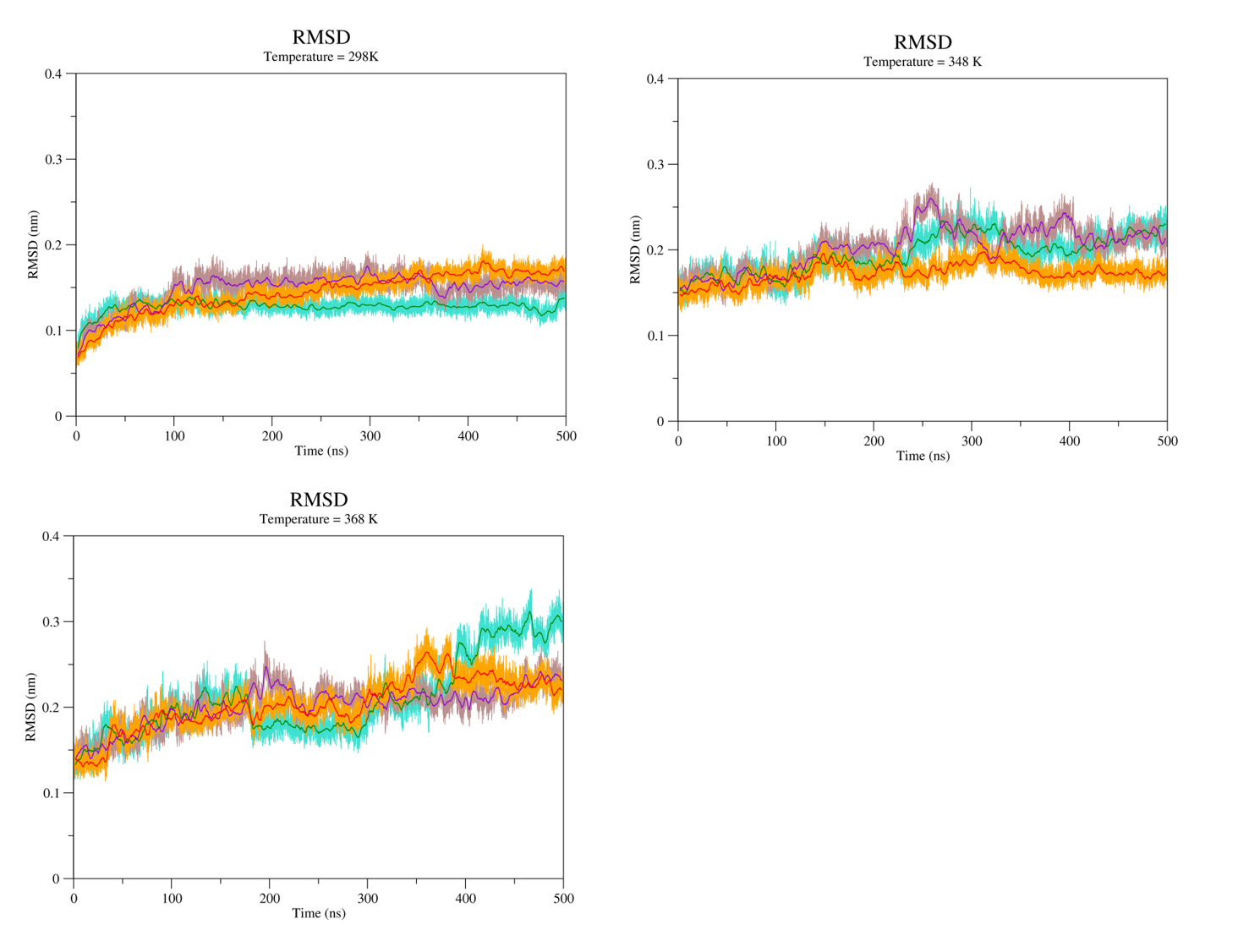


**Figure S8.** Root mean square deviation (RMSD) plots from 500ns MD simulations of cRML60-S2 in aqueous conditions at 298 K, 348 K, and 368 K, respectively. Three replicates were done at each temperature. The running average of each of the replicates is also included.


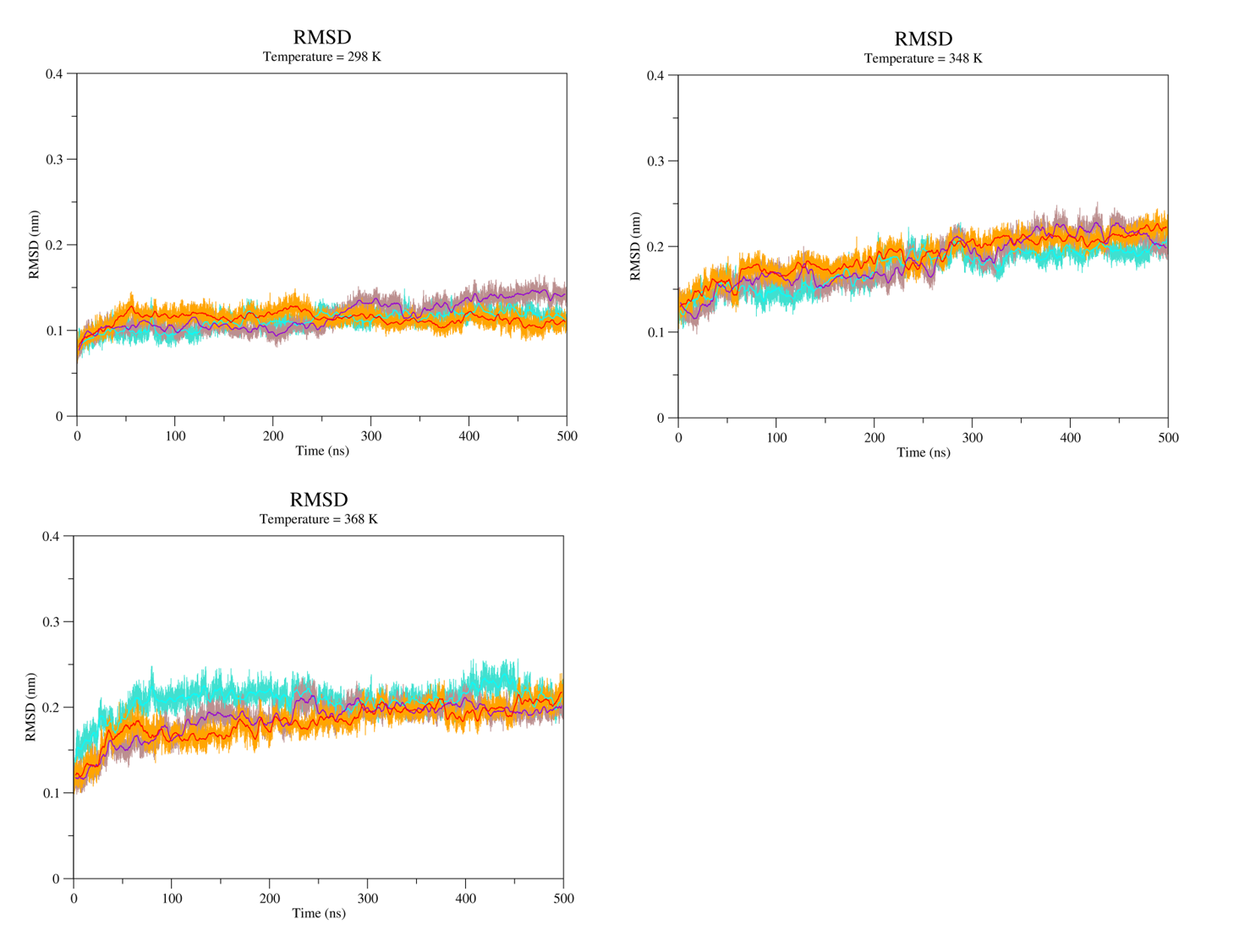


**Figure S9.** Root mean square deviation (RMSD) plots from 500 ns MD simulations of cRML60-S7 in aqueous conditions at 298 K, 348 K, and 368 K, respectively. Three replicates were done at each temperature. The running average of each of the replicates is also included.
